# Supplementary material for: Modeling glioblastoma heterogeneity as a dynamic network of cell states
Source: Mol Syst Biol. 2021 Sep 16;17(9):e10105. doi: 10.15252/msb.202010105 (PMC8444284; doi:10.15252/msb.202010105)
Supplement: Supplementary file 5 — Source Data for Figure 3 [file MSB-17-e10105-s001.zip › Figure3A_sourcedata/GSEA_3065/hallmarks_state5.GseaPreranked.1623416302988/HALLMARK_MYOGENESIS.html]

Details for gene set HALLMARK\_MYOGENESIS[GSEA]

|  || Dataset | state5 |
| Phenotype | NoPhenotypeAvailable |
| Upregulated in class | na\_pos |
| GeneSet | HALLMARK\_MYOGENESIS |
| Enrichment Score (ES) | 0.33861417 |
| Normalized Enrichment Score (NES) | 1.3398739 |
| Nominal p-value | 0.012987013 |
| FDR q-value | 0.172233 |
| FWER p-Value | 0.422 |
Table: GSEA Results Summary

  

Fig 1: Enrichment plot: HALLMARK\_MYOGENESIS      
 Profile of the Running ES Score & Positions of GeneSet Members on the Rank Ordered List

  

| PROBE | GENE SYMBOL | GENE\_TITLE | RANK IN GENE LIST | RANK METRIC SCORE | RUNNING ES | CORE ENRICHMENT || 1 | APP |  |  | 38 | 0.712 | 0.0358 | Yes |
| 2 | NCAM1 |  |  | 70 | 0.581 | 0.0651 | Yes |
| 3 | COL4A2 |  |  | 93 | 0.540 | 0.0931 | Yes |
| 4 | ITGB1 |  |  | 157 | 0.477 | 0.1129 | Yes |
| 5 | CLU |  |  | 162 | 0.474 | 0.1391 | Yes |
| 6 | ITGA7 |  |  | 207 | 0.436 | 0.1588 | Yes |
| 7 | AGRN |  |  | 271 | 0.398 | 0.1741 | Yes |
| 8 | SCD |  |  | 284 | 0.391 | 0.1948 | Yes |
| 9 | ATP6AP1 |  |  | 288 | 0.388 | 0.2163 | Yes |
| 10 | SPARC |  |  | 327 | 0.370 | 0.2329 | Yes |
| 11 | NOTCH1 |  |  | 348 | 0.361 | 0.2511 | Yes |
| 12 | ERBB3 |  |  | 411 | 0.337 | 0.2631 | Yes |
| 13 | IGFBP7 |  |  | 424 | 0.333 | 0.2805 | Yes |
| 14 | NAV2 |  |  | 478 | 0.318 | 0.2925 | Yes |
| 15 | PLXNB2 |  |  | 577 | 0.291 | 0.2978 | Yes |
| 16 | ADAM12 |  |  | 589 | 0.288 | 0.3128 | Yes |
| 17 | TSC2 |  |  | 703 | 0.261 | 0.3148 | Yes |
| 18 | AEBP1 |  |  | 825 | 0.237 | 0.3146 | Yes |
| 19 | PRNP |  |  | 851 | 0.232 | 0.3249 | Yes |
| 20 | EPHB3 |  |  | 1031 | 0.207 | 0.3164 | Yes |
| 21 | AKT2 |  |  | 1129 | 0.195 | 0.3164 | Yes |
| 22 | EIF4A2 |  |  | 1153 | 0.191 | 0.3246 | Yes |
| 23 | GAA |  |  | 1182 | 0.187 | 0.3320 | Yes |
| 24 | ITGB5 |  |  | 1216 | 0.183 | 0.3386 | Yes |
| 25 | ADCY9 |  |  | 1589 | 0.142 | 0.3048 | No |
| 26 | ITGB4 |  |  | 1681 | 0.135 | 0.3021 | No |
| 27 | KCNH2 |  |  | 1767 | 0.129 | 0.2998 | No |
| 28 | IGFBP3 |  |  | 2037 | 0.109 | 0.2757 | No |
| 29 | IFRD1 |  |  | 2073 | 0.107 | 0.2778 | No |
| 30 | SLC6A8 |  |  | 2268 | 0.094 | 0.2613 | No |
| 31 | PSEN2 |  |  | 2293 | 0.092 | 0.2638 | No |
| 32 | MEF2A |  |  | 2349 | 0.089 | 0.2626 | No |
| 33 | LPIN1 |  |  | 2453 | 0.082 | 0.2557 | No |
| 34 | SPEG |  |  | 2465 | 0.082 | 0.2590 | No |
| 35 | SPTAN1 |  |  | 2534 | 0.077 | 0.2557 | No |
| 36 | DMPK |  |  | 2541 | 0.077 | 0.2594 | No |
| 37 | SYNGR2 |  |  | 2623 | 0.073 | 0.2543 | No |
| 38 | COL6A2 |  |  | 2660 | 0.071 | 0.2543 | No |
| 39 | FDPS |  |  | 2849 | 0.060 | 0.2365 | No |
| 40 | SCHIP1 |  |  | 2911 | 0.057 | 0.2329 | No |
| 41 | DMD |  |  | 2914 | 0.057 | 0.2359 | No |
| 42 | MAPK12 |  |  | 3150 | 0.044 | 0.2119 | No |
| 43 | ABLIM1 |  |  | 3183 | 0.042 | 0.2107 | No |
| 44 | DTNA |  |  | 3219 | 0.040 | 0.2090 | No |
| 45 | REEP1 |  |  | 3264 | 0.038 | 0.2062 | No |
| 46 | STC2 |  |  | 3306 | 0.036 | 0.2036 | No |
| 47 | TGFB1 |  |  | 3503 | 0.027 | 0.1831 | No |
| 48 | AGL |  |  | 3509 | 0.027 | 0.1841 | No |
| 49 | SIRT2 |  |  | 3589 | 0.024 | 0.1765 | No |
| 50 | SH2B1 |  |  | 3795 | 0.016 | 0.1544 | No |
| 51 | SOD3 |  |  | 4105 | 0.003 | 0.1198 | No |
| 52 | SORBS1 |  |  | 4146 | 0.001 | 0.1153 | No |
| 53 | KIFC3 |  |  | 4580 | -0.017 | 0.0676 | No |
| 54 | SSPN |  |  | 4582 | -0.017 | 0.0685 | No |
| 55 | ACSL1 |  |  | 4862 | -0.028 | 0.0387 | No |
| 56 | MEF2C |  |  | 4905 | -0.029 | 0.0356 | No |
| 57 | SVIL |  |  | 4953 | -0.031 | 0.0321 | No |
| 58 | HDAC5 |  |  | 5064 | -0.035 | 0.0217 | No |
| 59 | GADD45B |  |  | 5106 | -0.037 | 0.0192 | No |
| 60 | CHRNB1 |  |  | 5205 | -0.041 | 0.0104 | No |
| 61 | PDE4DIP |  |  | 5468 | -0.051 | -0.0162 | No |
| 62 | MAPRE3 |  |  | 5485 | -0.052 | -0.0150 | No |
| 63 | FGF2 |  |  | 5527 | -0.054 | -0.0166 | No |
| 64 | EFS |  |  | 5531 | -0.054 | -0.0139 | No |
| 65 | MRAS |  |  | 5658 | -0.060 | -0.0246 | No |
| 66 | TPM2 |  |  | 5660 | -0.060 | -0.0214 | No |
| 67 | RIT1 |  |  | 5824 | -0.066 | -0.0360 | No |
| 68 | BHLHE40 |  |  | 5950 | -0.072 | -0.0460 | No |
| 69 | TEAD4 |  |  | 6250 | -0.087 | -0.0747 | No |
| 70 | SORBS3 |  |  | 6338 | -0.092 | -0.0793 | No |
| 71 | PFKM |  |  | 6344 | -0.092 | -0.0746 | No |
| 72 | MYO1C |  |  | 6459 | -0.099 | -0.0818 | No |
| 73 | OCEL1 |  |  | 6499 | -0.101 | -0.0805 | No |
| 74 | PKIA |  |  | 6634 | -0.110 | -0.0894 | No |
| 75 | TAGLN |  |  | 6733 | -0.116 | -0.0939 | No |
| 76 | BIN1 |  |  | 6777 | -0.119 | -0.0920 | No |
| 77 | FLII |  |  | 6817 | -0.122 | -0.0895 | No |
| 78 | CRAT |  |  | 6922 | -0.131 | -0.0938 | No |
| 79 | VIPR1 |  |  | 7051 | -0.142 | -0.1003 | No |
| 80 | MYLK |  |  | 7203 | -0.155 | -0.1085 | No |
| 81 | FKBP1B |  |  | 7211 | -0.156 | -0.1004 | No |
| 82 | MYH9 |  |  | 7248 | -0.161 | -0.0954 | No |
| 83 | BAG1 |  |  | 7474 | -0.189 | -0.1100 | No |
| 84 | GSN |  |  | 7578 | -0.201 | -0.1103 | No |
| 85 | PDLIM7 |  |  | 7864 | -0.247 | -0.1285 | No |
| 86 | FHL1 |  |  | 8198 | -0.326 | -0.1476 | No |
| 87 | CRYAB |  |  | 8315 | -0.358 | -0.1404 | No |
| 88 | WWTR1 |  |  | 8384 | -0.383 | -0.1265 | No |
| 89 | CKB |  |  | 8448 | -0.407 | -0.1106 | No |
| 90 | MYL6B |  |  | 8477 | -0.423 | -0.0899 | No |
| 91 | TPM3 |  |  | 8487 | -0.428 | -0.0668 | No |
| 92 | NQO1 |  |  | 8588 | -0.479 | -0.0510 | No |
| 93 | GABARAPL2 |  |  | 8658 | -0.531 | -0.0289 | No |
| 94 | CDKN1A |  |  | 8679 | -0.545 | -0.0004 | No |
| 95 | CNN3 |  |  | 8752 | -0.613 | 0.0261 | No |
Table: GSEA details [plain text format]

  

Fig 2: HALLMARK\_MYOGENESIS: Random ES distribution      
 Gene set null distribution of ES for **HALLMARK\_MYOGENESIS**
